# Comparative In Vitro Study between Biocompatible Chitosan-Based Magnetic Nanocapsules and Liposome Formulations with Potential Application in Anti-Inflammatory Therapy

**DOI:** 10.3390/ijms25158454

**Published:** 2024-08-02

**Authors:** Gabriela Vochița, Anca Niculina Cadinoiu, Delia-Mihaela Rață, Leonard Ionuț Atanase, Marcel Popa, Athar Mahdieh, Cosmin-Teodor Mihai, Alexandru-Bogdan Stache, Cristina-Veronica Moldovan, Elena Simona Băcăiţă, Iustina Petra Condriuc, Daniela Gherghel

**Affiliations:** 1 Institute of Biological Research Iasi, Branch of NIRDBS, 700107 Iasi, Romania; gabrielacapraru@yahoo.com (G.V.); daniela_gherghel@yahoo.com (D.G.); 2 Faculty of Medicine, Apollonia University of Iasi, 700511 Iasi, Romania; jancaniculina@yahoo.com (A.N.C.); iureadeliamihaela@yahoo.com (D.-M.R.); marpopa2001@yahoo.fr (M.P.); 3 Academy of Romanian Scientists, 050045 Bucharest, Romania; 4 Department of Pharmaceutics, School of Pharmacy, University of Oslo, Blindern, P.O. Box 1068, N-0316 Oslo, Norway; athar.mahdieh@farmasi.uio.no; 5 Praxis Medical Investigations, 700376 Iasi, Romania; 6 Department of Molecular Genetics, Center for Fundamental Research and Experimental Development in Translational Medicine—TRANSCEND, Regional Institute of Oncology, 700483 Iasi, Romania; stache.bogdan@gmail.com; 7 Department of Biology, Faculty of Biology, Alexandru Ioan Cuza University of Iasi, Bd. Carol I, Nr. 11, 700506 Iasi, Romania; 8 Faculty of Machine Manufacturing and Industrial Management, Gheorghe Asachi Technical University of Iasi, D. Mangeron Bld. No. 73, 700050 Iasi, Romania; bsimona77@yahoo.com; 9 Faculty of Medicine, Grigore T. Popa University of Medicine and Pharmacy, 700115 Iasi, Romania; iustina_condriuc@yahoo.com

**Keywords:** peptides, nanocapsules, liposomes, in vitro cytotoxicity, drug delivery

## Abstract

This study describes the comparison between the interaction of a series of peptide-functionalized chitosan-based nanocapsules and liposomes with two cell lines, i.e., mouse macrophages RAW 264.7 and human endothelial cells EA.hy926. Both types of nanocarriers are loaded with magnetic nanoparticles and designed for anti-inflammatory therapy. The choice of these magnetic nanostructures is argued based on their advantages in terms of size, morphology, chemical composition, and the multiple possibilities of modifying their surface. Moreover, active targeting might be ensured by using an external magnetic field. To explore the impact of chitosan-based nanocapsules and liposomes on cell cytophysiology, the cell viability, using the MTT assay, and cell morphology were investigated. The results revealed low to moderate cytotoxicity of free nanocapsules and significant cytotoxicity induced by chitosan-coated liposomes loaded with dexamethasone, confirming its release from the delivery system. Thus, after 48 h of treatment with nanocapsules, the viability of RAW 264.7 cells varied between 88.18% (OCNPM-1I, 3.125 µg/mL) and 76.37% (OCNPM-1, 25 µg/mL). In the same conditions, EA.hy926 cell viability was between 99.91% (OCNPM-3, 3.125 µg/mL) and 75.15% (OCNPM-3, 25 µg/mL) at the highest dose (25 µg/mL), the values being comparable for both cell lines. Referring to the cell reactivity after dexamethasone-loaded liposome application, the lowest viability of RAW 264.7 cells was 41.25% (CLDM5CP-1, 25 µg/mL) and 58.20% (CLDMM2CP-1 1.25 µg/mL) in the endothelial cell line, proving a selective character of action of nanocarriers. The cell morphology test, performed to support and confirm the results obtained by the MTT test, revealed a differentiated response for the two types of nano-carriers. As expected, an intense cytotoxic effect in the case of dexamethasone-loaded liposomes and a lack of cytotoxicity for drug-free nanocapsules were noticed. Therefore, our study demonstrated the biocompatible feature of the studied nanocarriers, which highlights them for future research as potential drug delivery systems for pharmacological applications, including anti-inflammatory therapy.

## 1. Introduction

Over the past decade, the continuous development of nanotechnology has led to improved diagnostic and treatment methods for many diseases [1,2,3]. The advantage of using nanomaterials in drug delivery systems (DDSs) resides in their specific physicochemical and biological properties (size, shape, and nature of the material), which make the pharmacodynamic and pharmacokinetic properties of incorporated drugs more effective and ensure their controlled and targeted release [4,5,6]. For effective targeting, the DDS must be maintained long enough in the physiological system to release the drug, but without being destroyed by the immune system [7]. This is obtained due to the properties of the nanocarriers, which increase the solubility and stability of the encapsulated drug, facilitating transmembrane transport and prolonging the release cycle [8]. There are two ways of targeted administration: intravascular drug delivery, in which nanoparticles enter the bloodstream through blood vessels and act intravascularly to treat specific diseases, and extravascular drug delivery, when nanoparticles cross the vascular endothelium and reach the target through local administration (subcutaneous, oral, inhalation). Nevertheless, the precise transport and delivery of drugs to the target area remains an important issue, which can be solved by additional research to make therapy more effective in various diseases.

One of the promising fields in which biomedical nanotechnology is successfully applied is in the diagnosis and treatment of inflammatory diseases [9,10,11,12,13,14,15]. Inflammation represents a physiological or pathological process in which the body tries to restore internal homeostasis in response to a series of stimuli, such as tissue injury or dysfunction, infection, stress, or hypoxia [16,17,18,19,20]. In inflammatory disease, cells with different functions are recruited to repair the injury or eliminate the infection, and B and T cells, by activating humoral or cellular immunity, specifically identify the aggressors, ensuring immune protection [21,22]. While moderate inflammatory response is helpful, excessive inflammatory response can cause systemic inflammatory syndrome, explosive release of cytokines [23,24], and multiple organ damage [25,26], even causing death [27].

In order to obtain nanocarriers able to encapsulate labile or toxic substances, various colloidal systems are developed, with polymer nanocapsules occupying a predominant place. These are vesicular systems composed of a core, mostly oily, surrounded by a wall with a polymeric structure [28]. With the help of these nanostructures, numerous features are improved: the enhancement of the dissolution process of natural or synthetic compounds, the intensification of the therapeutic index, and controlled transport and release, ensuring protection against photo-oxidation and chemical degradation [29,30]. They also improve the bioavailability of the drug, reducing possible side effects, and can reach the target organs with increased efficiency due to the small particle size, large surface area, the presence of Brownian motion, as well as the surface functionality of the nanocapsules, which could provide a greater cytotoxic effect even at low doses of therapeutic agents [31]. Nanocapsules used in drug delivery systems have a number of benefits compared to other nanocarriers; they present an extremely efficient encapsulation of active substances that can be incorporated into the core (which acts as a reservoir) or covalently attached/adsorbed to the polymer shell [32,33,34]. For the design of biomaterials with a high degree of synthesis and low impact on the environment, special attention was directed to natural sources of biopolymers, of which chitosan, cellulose, and alginate are the most used natural polymers in nanomedicine due to their size and geometry, large surface area, mechanical and barrier properties, lack of toxicity, biocompatibility, and biodegradability [35,36,37].

Other promising therapeutic nanocarriers are represented by liposomes, which, due to their major properties—biocompatibility and biodegradability—are widely used in anti-inflammatory, antiviral, and antitumor therapies [38,39,40,41,42].

The phospholipid-based structure, which forms a phospholipid bilayer in aqueous solution, allows encapsulation of both water-soluble and lipophilic compounds, protects the drugs from hydrolysis, and prolongs the biological half-life [43,44,45]. In addition, liposomes coated with a positive surface are characterized by an increased degree of stability, efficient targeting, and sustained release of drugs, this specificity enhancing the interaction of these nanocarriers with the cell surface [46,47]. At present, there are numerous strategies to functionalize conventional liposomes [48,49]. Among these strategies is the use of natural polymers, a special place being occupied by chitosan, a cationic polymer endowed with many unique biological properties, such as bioadhesivity, biocompatibility, biodegradability and low toxicity, and a high degree of adhesiveness, which favors the absorption of the drugs [50,51]. Therefore, protecting liposomes with chitosan coating to form a positive layer around negatively charged liposomes is an important approach to improve stability in various biological fluids, increase cellular uptake, and prolong drug release rate. Also, positively charged liposomes could interact with the cell membrane, allowing the opening of tight junctions and enhancing drug penetration [52,53,54].

Besides the studies regarding the improvement of the physicochemical characteristics of the nanocarriers (physical stability, size, drug loading efficiency), there is an increased concern for in vitro cytotoxicity studies [55].

In this context, the aim of the present study was to determine the biocompatibility degree of some newly synthetized magnetic nanocapsules based on chitosan, poly (NVPAI) and peptides, and also some magnetic cationic liposomes coated with chitosan and functionalized with peptides, loaded or not with dexamethasone, in order to identify their suitability in anti-inflammatory therapy. The original approach in this study is represented by the development of two types of complex nanoparticles (liposomes and nanocapsules) functionalized with two peptides and containing magnetic nanoparticles to be used in the treatment of anti-inflammatory diseases.

## 2. Results and Discussion

### 2.1. Nanocarrier Physicochemical Characterization

#### 2.1.1. Nanocapsules

The mean diameter and the zeta potential values are provided in Table 1. The average diameter of the nanocapsules in distilled water varied between 923 nm and 1127 nm. It was found that the nanocapsules that have magnetic nanoparticles (MNPs) in the polymer membrane have a larger diameter than the nanocapsules that contain MNPs in the core. Also, the presence of nanoparticles in the polymer membrane led to a decrease in the value of the zeta potential.

**Figure 1:**
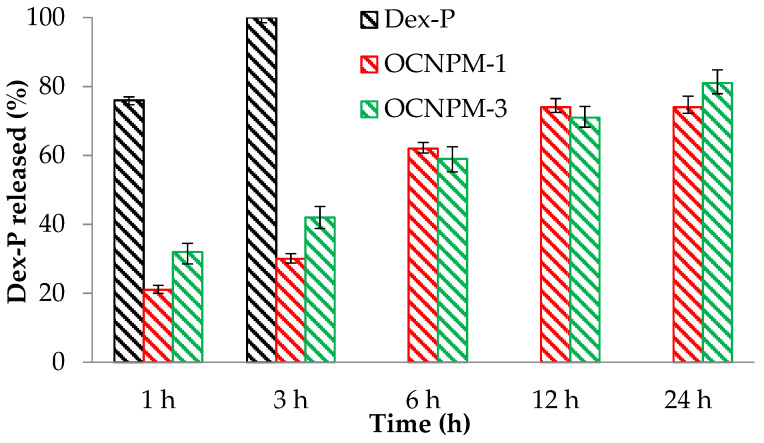
Release efficiency results of free Dex-P and Dex-P from nanocapsules in PBS solution with pH = 7.4.

Figure 1 shows the results of the release efficiency of dexamethasone phosphate (Dex-P) and Dex-P from nanocapsules in PBS solution with pH = 7.4 over a period of 24 h. According to the obtained results, it was found that the free drug was released in a proportion of 100% after 3 h. The release efficiency of Dex-P from OCNPM-1-type nanocapsules was approximately 74%, while for the OCNPM-3 sample, the release efficiency after 24 h was 81%. These results indicate the efficiency of nanocarriers to deliver the drug in a controlled and sustained manner.

#### 2.1.2. Liposomes

Magnetic cationic liposomes coated with chitosan and functionalized with peptides were obtained to be used in the therapy of inflammatory diseases of the ear. The average size of the liposomes, the zeta potential, and the drug encapsulation degree were determined, and the values obtained are detailed in Table 2.

The size of the liposomes varied between 260 nm and 307 nm, being slightly influenced by the lipid composition of the liposomes but also by the amount of incorporated MNPs. The values of the zeta potential in PBS (pH 7.4) were between +16 and +27 mV; these positive values are due, on the one hand, to chitosan and chitosan functionalized with peptides that was used for liposome coating, and on the other hand, to the magnetic particles incorporated in the aqueous core or in the lipid membrane.

To evaluate the release of dexamethasone phosphate from liposomes in an environment that simulates the blood environment, a phosphate buffer solution (PBS) with a pH of 7.4 was used. This experiment was carried out under constant conditions of temperature and agitation (37 °C and 50 rpm) using a dissolution apparatus equipped with a sampling station that helped to obtain a release curve up to 24 h.

**Figure 2:**
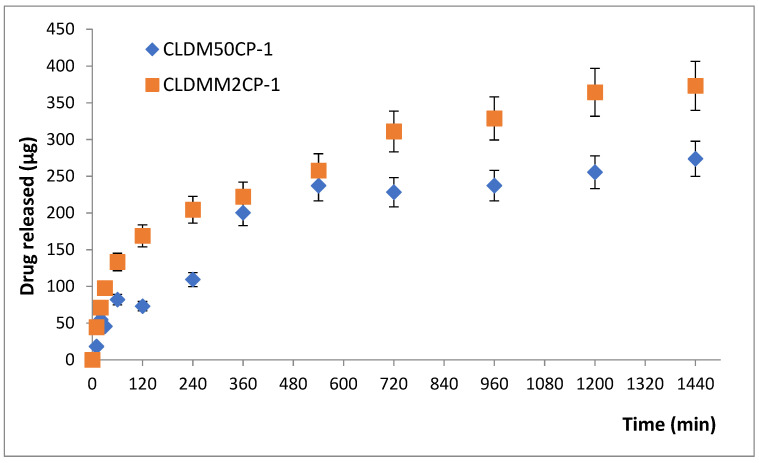
The amount of drug released (µg) from magnetic cationic liposomes coated with chitosan, up to 24 h.

Figure 2 shows the release kinetics of dexamethasone phosphate for the samples CLDM5CP-1 and CLDMM2CP-1. The two samples have the same lipid composition, but the amount of incorporated MNPs differs as well as the way of incorporation; for the sample CLDM5CP-1, the MNPs were introduced into the drug solution, and for the sample CLDMM2CP-1, they were introduced into the lipid solution. The amount of drug released after 24 h for the sample CLDMM2CP-1 was 373 µg, higher than that recorded for the sample CLDM5CP-1, which was 274 µg. The incorporation of MNPs into the lipid membrane can lead to a decrease in its stability, and therefore, the drug from the aqueous core can be released a little faster.

### 2.2. In Vitro Cell Viability Determination

In vitro studies on RAW 264.7 mouse macrophages and EA.hy926 human endothelial cells were used to investigate the degree of toxicity of four nanocapsules and five liposome samples, using a wide range of doses, namely 3.125, 6.25, 12.5, and 25 µg/mL.

**Figure 3:**
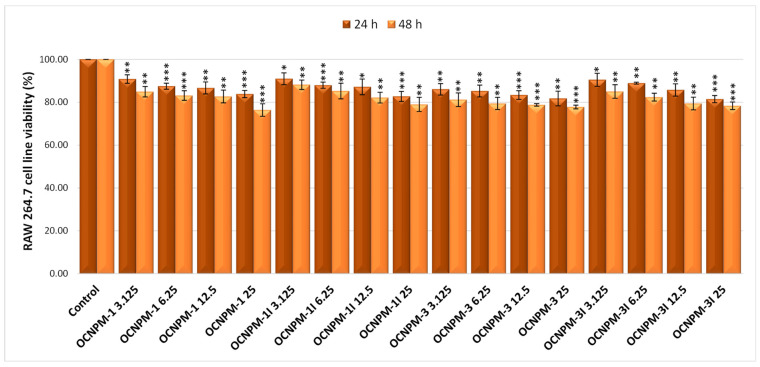
Effect of 24 and 48 h treatment with different concentrations of chitosan-based nanocapsules with magnetic particles on the viability of RAW 264.7 mouse macrophages. * *p* < 0.05, ** *p* < 0.01, and *** *p* < 0.001.

For the RAW 264.7 cells, the 24 h treatment, for all studied nanocapsules, induced a non-cytotoxic effect on cell viability, ranging from 86.05% (OCNPM-3) to 90.97% (OCNPM-1I) for the lowest dose (3.125 µg/mL), and from 81.46% (OCNPM-3I) to 83.84% (OCNPM-1) for the maximum tested dose (25 µg/mL), compared to the untreated control, considered 100%. In the case of 48 h treatment, with the previously mentioned nanocapsules, a negligible impact on cell viability was detected, in a dose-dependent manner, which varies from 81.18% (OCNPM-3) to 88.18% (OCNPM-1I) for the lowest dose (3.125 µg/mL), as well as a weak cytotoxic effect, the percentage of viability being from 76.37% (OCNPM-1) to 78.99% (OCNPM-1I) for the maximum dose (25 µg/mL), compared to the untreated control (100%), as shown in Figure 3.

**Figure 4:**
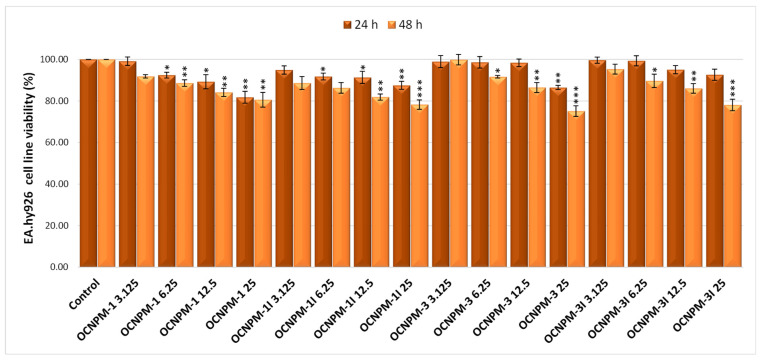
Effect of 24 and 48 h treatment with different concentrations of chitosan-based nanocapsules with magnetic particles on the viability of EA.hy926 human endothelial cells. * *p* < 0.05, ** *p* < 0.01, and *** *p* < 0.001.

In the case of EA.hy926 human endothelial cells, the 24 h treatment with magnetic nanocapsules determined an insignificant effect on cell viability for the smallest dose (3.125 µg/mL), the values being very close to those of the untreated control. For the highest dose (25 µg/mL), we registered values of cell viability ranging from 81.81% (OCNPM-1) to 92.65% (OCNPM-3I) compared to the untreated control (100%). The 48 h treatment with the magnetic nanocapsules was followed by slight decreases in cell viability values, ranging between 88.66% (OCNPM-1I) and 99.91% (OCNPM-3) for the dose of 3.125 µg/mL, and between 75.15% (OCNPM-3) and 80.61% (OCNPM-1) for the dose of 25 µg/mL, as illustrated in Figure 4.

A greater reactivity is found in the case of RAW 264.7 mouse macrophages when treated with magnetic nanocapsules, there being no noticeable differences between those loaded with magnetic particles in the capsule core compared to those embedded in the membrane.

In vitro studies on RAW 264.7 mouse macrophages and EA.hy926 human endothelial cells also included a series of magnetic cationic liposomes loaded with dexamethasone and coated with peptide-functionalized oligochitosan.

**Figure 5:**
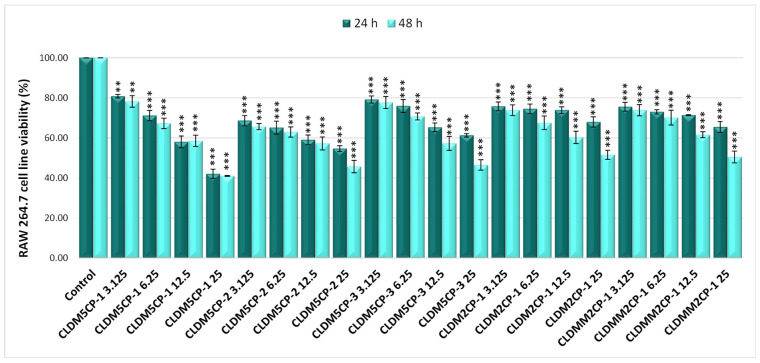
Effect of 24 and 48 h treatment with magnetic cationic liposomes, in different concentrations, on the viability of RAW 264.7 mouse macrophages. ** *p* < 0.01 and *** *p* < 0.001.

As seen in Figure 5, the treatment with these magnetic liposomes applied to RAW 264.7 cells induced a notable impact on cell viability, even after 24 h, at the dose of 3.125 µg/mL, with recorded values being from 68.63% (CLDM5CP-2) to 80.84% (CLDM5CP-3) and from 42.10% (CLDM5CP-1) to 68.00% (CLDM2CP-1) at the dose of 25 µg/mL. The 48 h treatment resulted in a stronger impairment of the viability of RAW 264.7 cells under the action of magnetic liposomes loaded with dexamethasone, the percentage values of cell viability being between 65.65% (CLDM5CP-2) and 78.23% (CLDM5CP-1) at the dose of 3.125 µg/mL, as well as between 41.05% (CLDM5CP-1) and 51.61% (CLDM2CP-1) at the highest dose (25 µg/mL).

**Figure 6:**
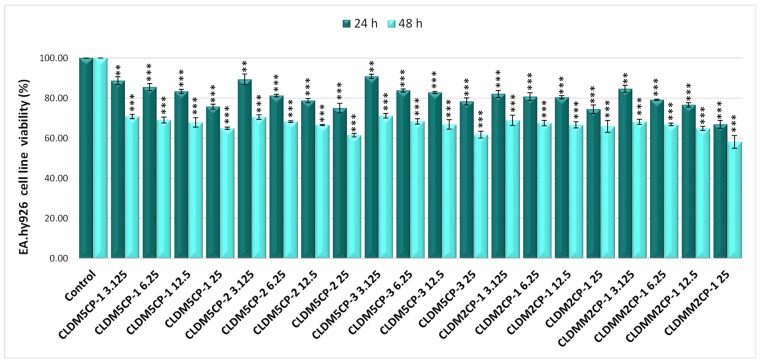
Effect of 24 and 48 h treatment with magnetic cationic liposomes, in different concentrations, on the viability of EA.hy926 human endothelial cells. ** *p* < 0.01 and *** *p* < 0.001.

Less obvious decreases in cell viability were found after treatment with magnetic liposomes in the case of EA.hy926 human endothelial cells. The lowest values were evidenced for the sample CLDMM2CP-1 (66.94%) at the dose of 25 µg/mL, both after the 24 h and after the 48 h treatment (58.20%), as shown in Figure 6.

According to our results and international criteria [56,57], it can be concluded that nanocapsules based on chitosan, poly (NVPAI), and peptides, loaded with magnetic particles, are weakly cytotoxic (60–80% viability) in the case of both tested cell lines.

There is some differentiation for magnetic cationic liposomes loaded with dexamethasone and coated with peptide-functionalized oligochitosan in the sense that on RAW 264.7 cells, CLDM5CP-1, CLDM5CP-2, and CLDM5CP-3 samples show a moderate cytotoxic effect (40–60% viability), whereas CLDM2CP-1 and CLDMM2CP-1 samples are slightly cytotoxic (60–80% viability), with the exception of the CLDMM2CP-1 sample, which is moderately cytotoxic (40–60% viability); this effect is also noted on EA.hy926 cells.

Our results are in agreement with those provided in the literature. Thus, polymeric nanoparticles based on chitosan are excellent drug carriers due to their non-toxicity, biodegradability, bioactivity, and specific targeting triggered by their cationic nature [58,59]. Cell viability studies on murine 4T1 breast cancer and CT26 colon cancer cells indicated the low cytotoxicity of RDG NPs, with the cell viability remaining approximately 80% [60]. Other studies proved that blank hyaluronic acid-coated chitosan/gelatin nanoparticles have no cytotoxicity effect, the cell viability being higher than 90% even at the highest concentration tested on the A-375 melanoma cell line [61]. Likewise, the components of cationic dexamethasone palmitate nanoparticles did not show a toxic effect on RAW 264.7 macrophages [62]. Cytotoxicity tests of chitosan-based nanocapsules on Vero cells demonstrated that grafting with poly(ethylene glycol) (PEG) induced less toxicity in the cells, especially at high concentrations, being a promising strategy to modulate the cytotoxicity [63]. Empty polyelectrolyte nanocapsule systems composed of biopolymers, chitosan, and heparin (CS–HP) showed no toxicity on MCF-7 cells, even at higher concentrations, which proved their biocompatible nature [64]. The poly(N-Acryloyl L-Leucine methyl ester) (pLME) nanocapsules with a hollow core (pLME) did not provoke nuclear/cellular damage in RAW 267.4 macrophages, expressed by increased levels of viability, remaining safe for cells [65].

Similar manifestations were found in the case of various liposome formulations. Flexible liposomes (FLs) coated with chitosan (CS) did not show notable cytotoxicity on HT29 colon cancer cells, with more than 94% of cells surviving [40]. In vitro biocompatibility assays performed with blank liposomes and hybrid systems (liposomes and thermosensitive hydrogel) on BALB/3T3 fibroblasts revealed a minor reduction in cell viability due to blank liposome exposure, with the hybrid system showing no cytotoxicity after 24 h of incubation, which suggests the absence of toxicity in the formulation [66]. The viability of Huh-7 (human hepatoma cells) and ACE2 + A549 (A549 lung carcinoma cells expressing human ACE2, angiotensin-converting enzyme 2) cells was not affected when exposed to empty liposomes, used within lactoferrin–liposome complexes, for antiviral treatment [39]. The plain chitosan-coated nanoliposomes displayed a reduced % cytotoxicity on MCF-7 cells compared with those loaded with two lipophilic agents, exemestane (EXE) and genistein (GEN), systems used in breast cancer therapy [53].

### 2.3. IC_50_ Calculation

According to the MTT results, the IC_50_ values obtained after 24 h of treatment, applied to the RAW 264.7 cell line with the chitosan-based nanocapsules, ranged from 70.97 µg/mL (OCNPM-3I) to 76.662 µg/mL (OCNPM-3). The differences between the four nanocarriers were insignificant (Table 3). It is worth noting the very close IC_50_ values obtained after the two treatment periods in the case of the OCNPM-3I nanocapsules, allowing us to assume that the weak cytotoxic effect is induced during the first 24 h of treatment. This response is an important advantage because it can ensure an increased retention of this nanocarrier in the body, without causing unwanted cytotoxic effects, as a result of extending the duration of exposure. In the case of EA.hy926 cells, the 24 h treatment with magnetic nanocapsules led to IC_50_ values between 63.22 µg/mL (OCNPM-1) and 83.73 µg/mL (OCNPM-3I). In general, the IC_50_ values were lower after the 48 h treatment with nanocapsules than those recorded after 24 h.

Regarding the magnetic cationic liposomes, the IC_50_ values, after 24 h of treatment, were between the limits of 24.46 µg/mL (CLDM5CP-1) and 59.68 µg/mL (CLDM2CP-1) in RAW 264.7 macrophages and between 47.97 µg/mL (CLDMM2CP-1) and 66.47 µg/mL (CLDM2CP-1) for the EA.hy926 cell line, as seen in Table 4. After 48 h of treatment with cationic magnetic liposomes, in general, similar or slightly lower values of the IC_50_ index were found, compared with the 24 h interval.

Typically, the cellular response to treatments with increasing doses of drugs loaded in various types of nanostructures is dose-dependent, a manifestation observed in our results, being in accordance with the consulted literature. Studies using two lines of acute lymphoblastic leukemia (ALL) origin, RS4;11 and Nalm6, showed progressive dose-dependent decreases (10 nM to 10 μM) in cell viability (10–30%) after Dex-NP treatment (assembled from an amphiphilic block copolymer of poly(ethylene glycol) (PEG) and poly(ε-caprolactone) (PCL) bearing suspended cyclic ketals (ECT2). The response was in direct relation to the low IC50 values, 2.4 nM for RS4;11, *p* = 0.16 and 5.08 nM for Nalm6, *p* = 0.6 nM [67]. Furthermore, testing the cytotoxic activity of dexamethasone encapsulated in Poly (d, l- lactide-co-glycolide) (PLGA) (MS)—PLGA MS—on human hepatocellular carcinoma HepG2 revealed that treatment for 24 h with concentrations ranging from 10 μg/mL to 100 μg/mL leads to decreased values of cell viability in a dose-dependent manner, with an IC50 value of 68.5 mM, suggesting low cytotoxicity due to slower release in vitro for this time frame [68].

### 2.4. Cell Morphology Assay

A complementary objective of the present study was the evaluation of cytomorphological changes in response to the exposure of different newly synthesized nanocarriers.

**Figure 7:**
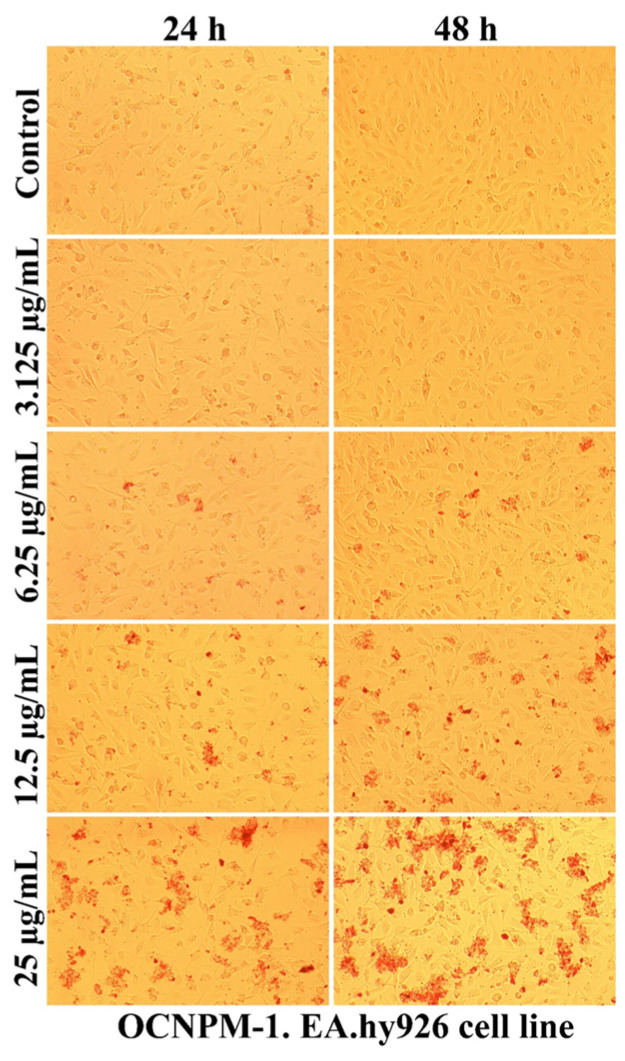
The morphology of the EA.hy926 cells after 24 and 48 h of OCNPM-1 treatment.

**Figure 8:**
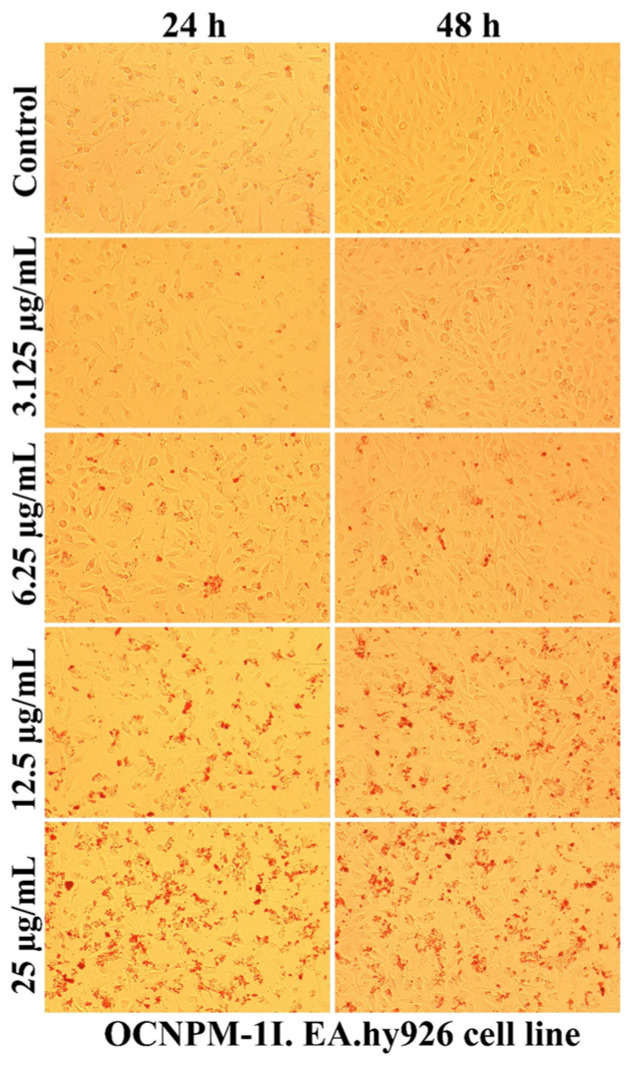
The morphology of the EA.hy926 cells after 24 and 48 h of OCNPM-1I treatment.

**Figure 9:**
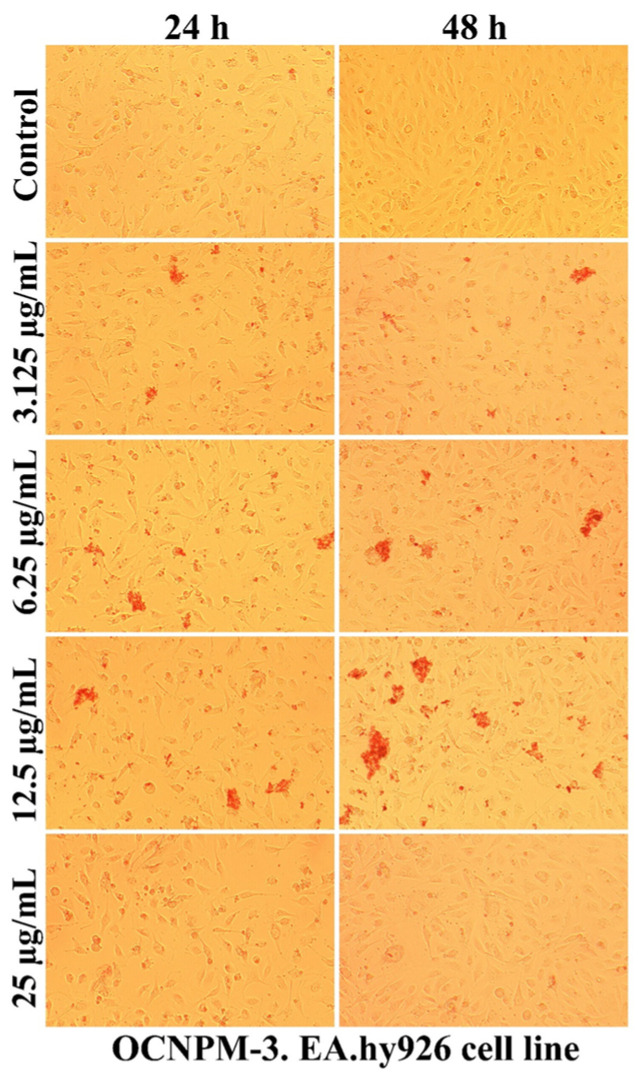
The morphology of the EA.hy926 cells after 24 and 48 h of OCNPM-3 treatment.

**Figure 10:**
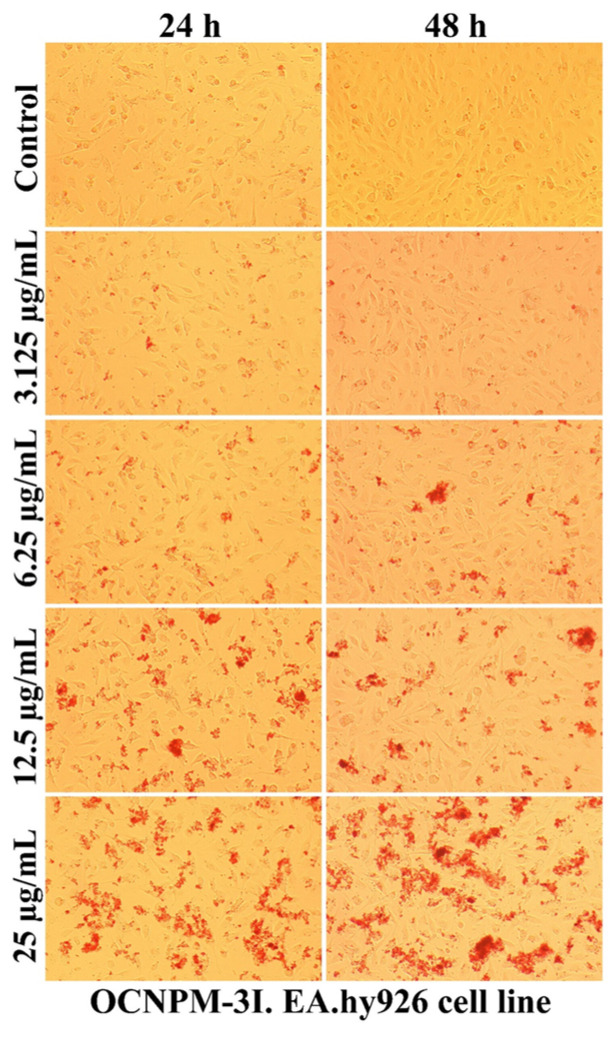
The morphology of the EA.hy926 cells after 24 and 48 h of OCNPM-3I treatment.

As can be seen in Figure 7, Figure 8, Figure 9 and Figure 10, the morphology of the cells undergoes transformations both depending on the concentration of the tested nanocarriers and the duration of the treatment. In general, the treatment decreases the ability of the cells to adhere to the substrate (well plates) for the formation of the cell monolayer. Also, there is an elongation and thinning of the pseudopods, in an attempt by the cells to restore the intercellular connections in the gapes resulting from the detachment of the affected cells. In the selected photomicrographs, the internalization of nanoparticles can be observed, the process being marked with red arrows. Moreover, it is found that the penetration of the nanocapsules through the cell membrane is more intense at the low used concentrations, because increasing the dose and prolonging the duration of the treatment facilitates the aggregation of these nanocarriers. Another process, visualized especially at the highest dose (25 µg/mL), was represented by the formation of vesicles inside the cells, denoting the existence of an endocytosis process.

The use of non-sonicated nanocapsules led to the formation inside cells of larger vesicles compared to the use of grafted nanocapsules, the response being proportional to the increased degree of toxicity. Also, the positive charge of polymers ensures the transport of molecules through the intercellular space specific to epithelial cells, this mechanism being regulated by tight junction complexes [69,70]. Moreover, the cytotoxicity of nanocapsules depends on the surface properties, and obtaining nanocapsules with a low-density coating of PEG molecules (5 nmol/mg) showed a high degree of internalization in Vero cells, a useful property for the potential application as drug reservoirs directed to cytoplasmic release [63].

**Figure 11:**
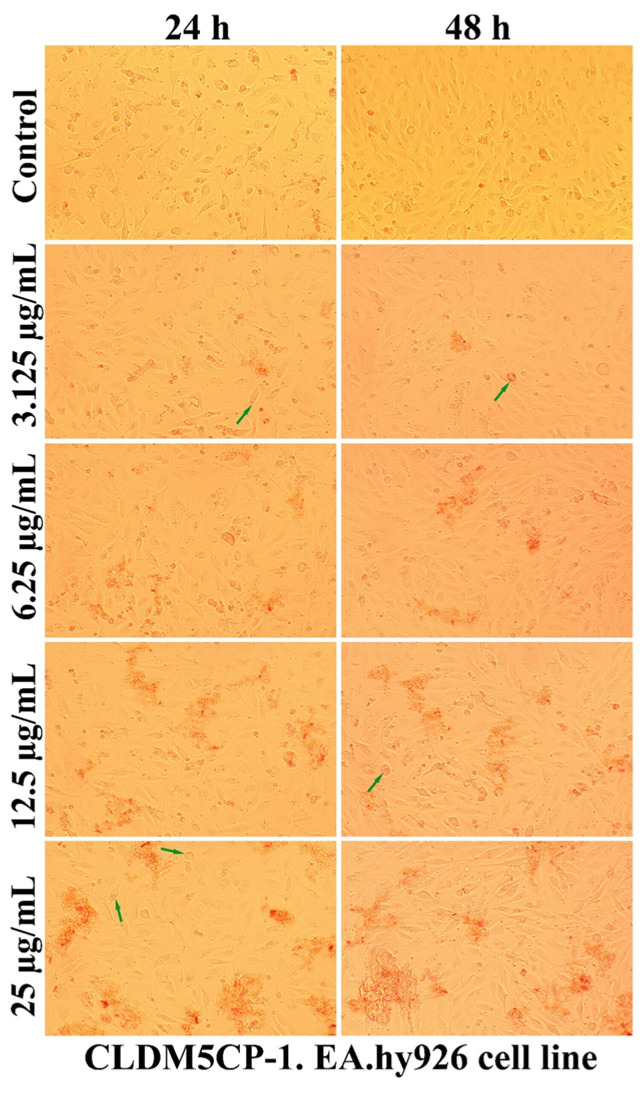
The morphology of the EA.hy926 cells after 24 and 48 h of CLDM5CP-1 treatment.

**Figure 12:**
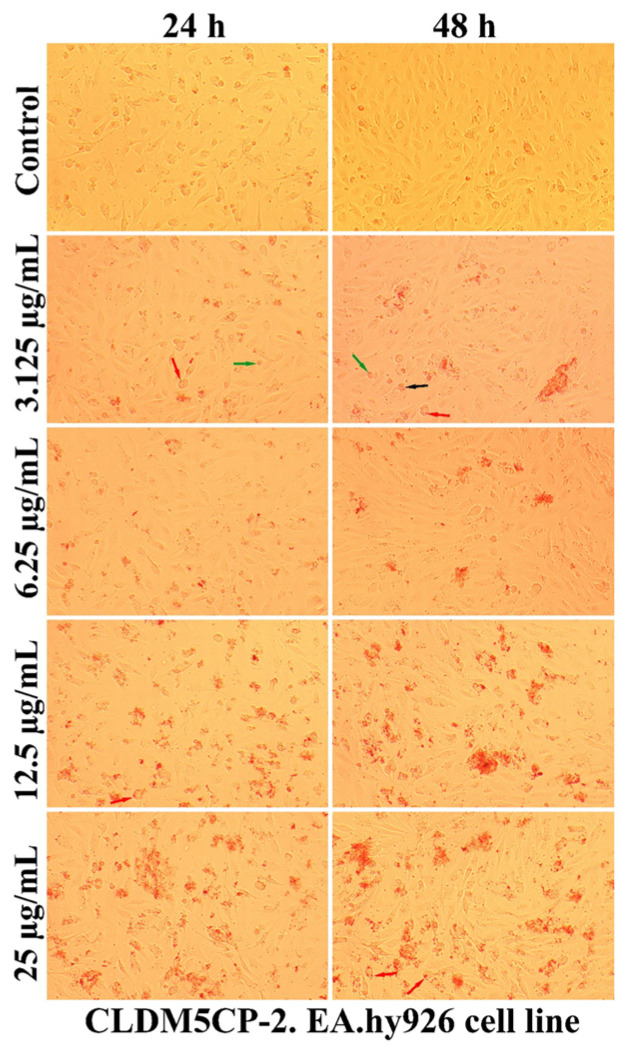
The morphology of the EA.hy926 cells after 24 and 48 h of CLDM5CP-2 treatment.

**Figure 13:**
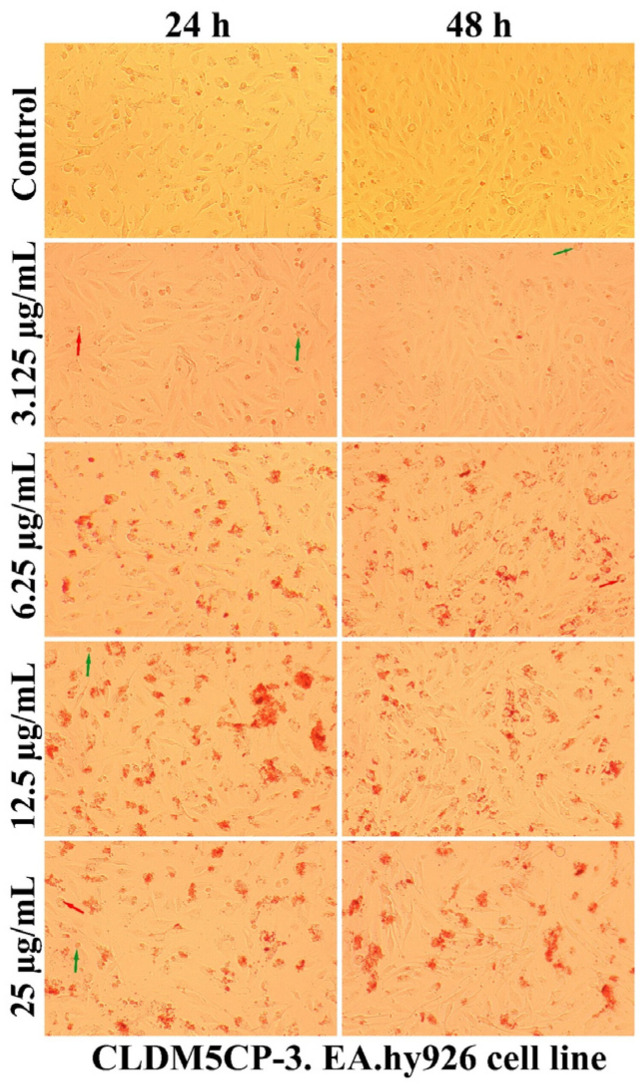
The morphology of the EA.hy926 cells after 24 and 48 h of CLDM5CP-3 treatment.

**Figure 14:**
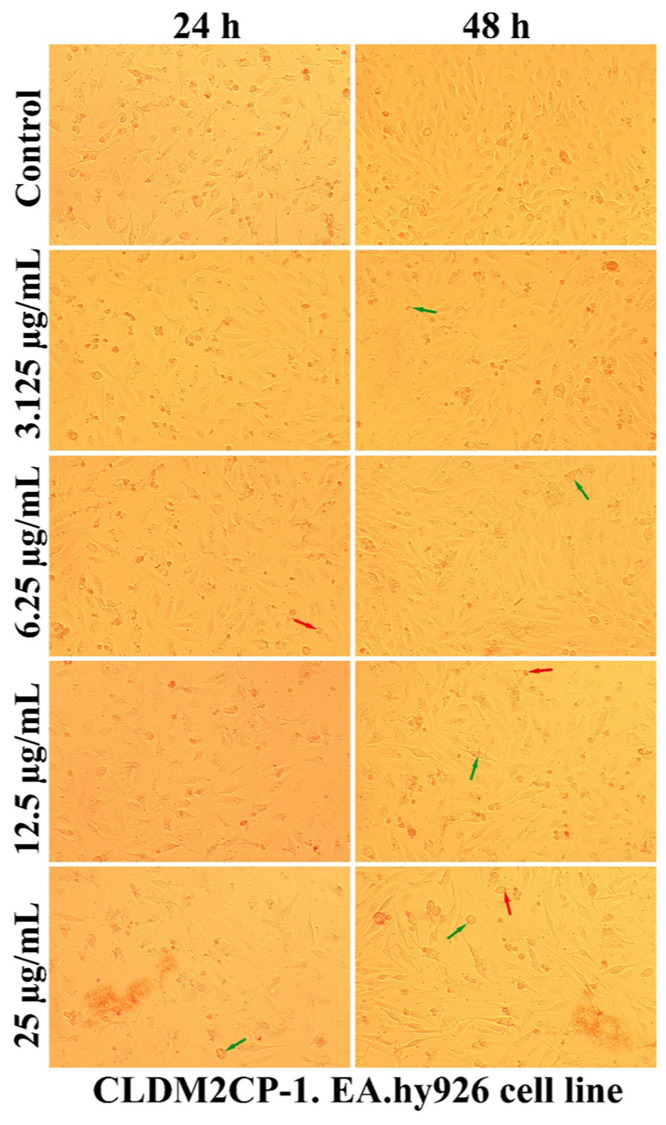
The morphology of the EA.hy926 cells after 24 and 48 h of CLDM2CP-1 treatment.

**Figure 15:**
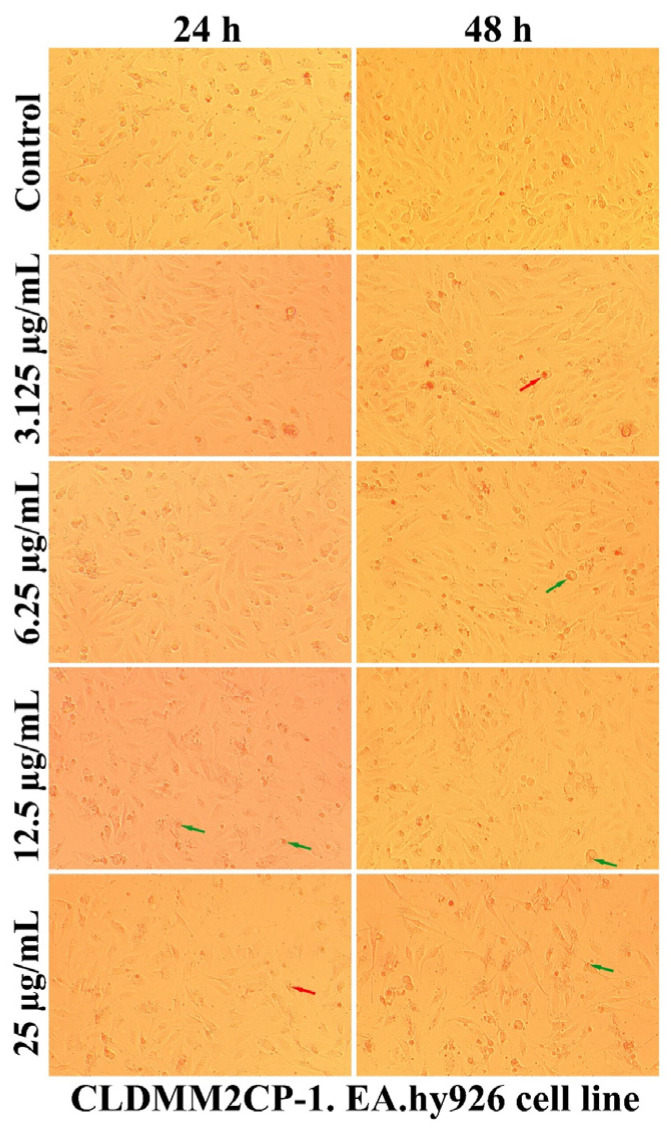
The morphology of the EA.hy926 cells after 24 and 48 h of CLDMM2CP-1 treatment.

A qualitative evaluation of the interaction of some newly synthesized liposomes with EA.hy296 cells was carried out by establishing the cell morphology, both after 24 and after 48 h of treatment. In Figure 11, Figure 12, Figure 13, Figure 14 and Figure 15, more or less intense phenomena can be visualized, embodied in changes in the integrity of the cell membrane, the formation of vesicles, supposed endocytosis (green arrows), and the identification of drop-like inclusions, proving the internalization of the tested liposomes (red arrows). Some cells showed membrane shrinkages and a reduction in the size of the nucleus, which may indicate the existence of an apoptotic process (black arrow). As in the case of nanocapsules, prolonging the treatment and increasing the concentration of the tested liposomes was accompanied by the formation of aggregations of different sizes around the cells.

Changing the physicochemical features of liposomes by chitosan coating causes the surface charge to change from negative to positive, which improves their interaction with cells and allows the use of chitosan for a large number of possible applications, not only limited to regenerative medicine. In addition, the cellular uptake was enhanced in the case of cells incubated with liposomes embedded in thermosensitive hydrogels and chitosan coating, compared to cells treated with liposomes alone [66]. Some liposomes obtained by microfluidic techniques and loaded with dexamethasone hemisuccinate showed non-toxic proprieties to human Adult Retinal Pigment Epithelial cell line-19 (ARPE-19) cells, and efficiently reduce inflammation after drug delivery [71]. Some nanostructures’ penetration efficiency through the cell membrane is influenced by the number and type of amino acids with which they can bind [72].

Considering the obtained results, it can be supposed that the effect of dexamethasone-loaded liposomes could be attributed to the triggering of apoptotic processes, signalled in the cell morphology tests. The specialized literature mentions that negatively charged liposomes appear to naturally target cells of the mononuclear phagocytic system (MPS), especially macrophages, most efficiently by interacting with scavenger receptors on their surface. The use of liposomes for specific targeting of monocytes/macrophages is not limited to drug delivery, but has a role in cell ablation and activation for the treatment of chronic inflammation, atherosclerosis, and cancer [73]. Studies on RAW 264.7 cells have argued that the mechanism of action of magnetic cationic liposomes is apoptotic, induced by liposome components. Thus, the cationic component stearylamine (SA) in liposomes generates reactive oxygen species (ROS) in macrophages, releasing cytochrome c, caspase-3 and -8, and activate protein kinase C (PKC) δ by cell surface proteoglycan interaction, and liposomal cholesterol accomplishes the association with macrophages. Also, the generation of ROS was decreased by coating the liposomes with polyethylene glycol (PEG), thus reducing the induction of apoptosis [74,75,76]. In addition, dexamethasone itself can induce apoptosis in RAW 264.7 macrophages by ROS generation and mitochondria dependence via Krüppel-like factor 9 (KLF9) [77]; in human colon cancer cell lines LoVo and HCT116, rich in glucocorticoid receptor α (GRα) protein, correlated with decreased NF-κB p65 activity [78]; and in thymocytes, due to early ceramide generation caused by the activation of an acidic sphingomyelinase (aSMase) [79].

### 2.5. Statistical Analysis

#### 2.5.1. Peptide-Functionalized Magnetic Nanocapsules

The factors that were modified in the design of peptide-functionalized magnetic nanocapsules and which in the following statistical analysis will be considered the independent variables are:

1. Molar ratio (MR) of NH_2_ groups of OCS/anhydride cycles of poly(NVPAI); it has two distinct values: 0.5/1 for OCNPM-1 and OCNPM-1I samples, and 1.5/1 for OCNPM-3 and OCNPM-3I samples, respectively.
2. Placement of magnetic nanoparticles (L), in two configurations: in the core of the capsules for OCNPM-1 and OCNPM-3 and in the membrane of the capsules for OCNPM-1I and OCNPM-3I.
3. Concentration of the nanocapsules (C) used for the cytotoxicity test; it has four distinct values of 3.125 µg/mL, 6.25 µg/mL, 12.5 µg/mL, 25 µg/mL.

The way in which these factors and their interactions impact the cell viability, at 24 and 48 h, for both cell lines, was evaluated through a three-way ANOVA [80], in which it is considered the dependent variable. The interpretation of the ANOVA results was based on the *p*-values obtained (see Table 5). In this analysis, *p*-values smaller than the established significance level, typically set at 0.05, indicate that the factor or interactions between factors have a statistically significant effect on the dependent variable, which in this study is cell viability.

The analysis of the independent variables reveals that the molar ratio of NH_2_ groups to anhydride cycles in the poly (NVPAI) structure, as well as the localization of magnetic nanoparticles (either in the core, or the membrane of the capsules) have a significant effect on cell viability at both 24 and 48 h across the cell types studied. This conclusion is supported by *p*-values of 0.002 and 0.015, which are substantially below the critical threshold of 0.05, indicating robust statistical significance. However, a notable exception is observed in the EA.hy926 cell line, where the placement of magnetic nanoparticles does not significantly affect cell viability at 24 h. The influence of nanoparticle localization in EA.hy926 cells becomes more pronounced and significant over time, emphasizing its delayed impact.

The *p*-value for drug concentration has a very small value, demonstrating that the concentration of the capsules has a highly significant influence on cell viability. This effect surpasses the impacts of both the molar ratio and the localization of magnetic nanoparticles across all observation times and cell lines analyzed.

In evaluating the interactions among the factors, the one between drug concentration and molar ratio impacts the cell viability, particularly at 48 h in both cell lines; this suggests that the combined impact of these factors intensifies over time. Additionally, the interaction between capsule concentration and the placement of magnetic nanoparticles is significant for the RAW 264.7 cell line at 24 h and the EA.hy926 cell line at 48 h. However, these interactions are either marginally significant or not significant at other time points, indicating that their effects are both cell-specific and time-dependent.

The interaction between the placement of magnetic nanoparticles and the molar ratio of the nanocapsules is significant for the RAW 264.7 cell line at 24 h, but loses significance after 48 h. This change indicates that the initial combined effects of these factors decrease over time, possibly due to cellular adaptation or the increasing influence of other factors. In contrast, for the EA.hy926 cell line, this interaction does not reach significance at either time point, although it approaches significance at 48 h, suggesting a potentially emerging influence. Notably, the three-way interaction among these factors is not significant, suggesting that cell viability is predominantly governed by the individual effects and the two-way interactions rather than their combined influence.

This comparative analysis underscores the necessity of factoring in both timing and specific cell type when developing and refining treatment strategies. For RAW 264.7 cells, optimizing the initial conditions of magnetic nanoparticle placement and molar ratio is crucial for early cell viability, as their interaction is significantly impactful at 24 h. Adjusting these parameters in the initial stages can therefore enhance early outcomes. Conversely, for EA.hy926 cells, the early stages show a lack of significant interaction between the effects of nanoparticle placement and molar ratio, suggesting that these factors can be addressed independently in initial treatment strategies. However, attention must be given to their potential interaction at later stages, as their combined effects may become more relevant over time.

#### 2.5.2. Peptide-Functionalized Magnetic Liposomes

In the case of the peptide-functionalized magnetic liposomes, the following factors were considered as independent variables:

1. the EPC:Chol:DOTAP mass ratio (MR) used in the experimental program, for which three distinct values were identified: 22:2:1 for CLDM5CP-1, CLDM2CP-1, and CLDMM2CP-1 samples, 21:2:2 for the CLDM5CP-2 sample, and 19:4:2 for the CLDM5CP-3 sample;
2. magnetic liposomes, including both the quantity of magnetic nanoparticles and the method of incorporation (MLs), with three variants: 12.5 mg in drug solution for CLDM5CP-1, CLDM5CP-2, and CLDM5CP-3 samples, 10 mg in drug solution for CLDM2CP-1, and 10 mg in lipid solution for the CLDMM2CP-1 sample;
3. the concentration of the lipids (C) in the liposome suspension, with four distinct values: 3.125 µg/mL, 6.25 µg/mL, 12.5 µg/mL, 25 µg/mL.

Following the approach of the previous analysis, a three-way ANOVA was conducted to evaluate the effects and interactions of these factors on cell viability at 24 and 48 h across both cell lines (Table 6). It is important to note that the available experimental data did not allow the evaluation of the interactions between the molar ratio and magnetic nanoparticles, or the combined effects of all three factors; therefore, these interactions were omitted in the following analysis.

Based on these results, it was concluded that, for the RAW 264.7 cell line, the molar ratio does not significantly impact cell viability at either 24 or 48 h. However, the method and quantity of magnetic nanoparticles have a significant effect on cell viability, with decreasing impact over 48 h, suggesting that the initial influence decreases over time. Drug concentration, on the other hand, remains highly significant throughout, as evidenced by its very low *p*-value, indicating a consistently strong effect on cell viability over time. Regarding factor interactions, there is no significant combined effect of lipid concentration and molar ratio on cell viability at either time point for RAW 264.7 cells. Conversely, the interaction between lipid concentration and the method of nanoparticle incorporation significantly influences cell viability at 24 h, but this effect is not sustained at 48 h, indicating that the impact is not long-lasting.

For the EA.hy926 cell line, the molar ratio significantly impacts cell viability at 24 h, indicating its critical role in the initial cellular response. However, this influence diminishes over time, suggesting that other factors or cellular adaptations mitigate its early effect. The method and dosage of the initial incorporation of magnetic nanoparticles are significant for cell viability, with sustained effects observed at both 24 and 48 h, as evidenced by *p*-values below 0.05 at both time points. Lipid concentration remains an important factor, with extremely low *p*-values at both time points, highlighting its critical and persistent influence on cell viability. In contrast, interactions between these factors do not significantly affect cell viability over time, indicating that their combined effects are not substantial.

The analysis highlights that while both cell lines exhibit a strong dependency on lipid concentration for cell viability, the impacts of molar ratio and magnetic nanoparticles differ. In the RAW 264.7 cell line, cell viability is initially influenced by MLs, but this dependency diminishes over time. Conversely, the EA.hy926 cell line demonstrates a sustained response to magnetic nanoparticles and a significant initial response to the molar ratio. These variations suggest cell-type-specific responses to these factors, emphasizing the need for tailored approaches in treatment design.

**Figure 16:**
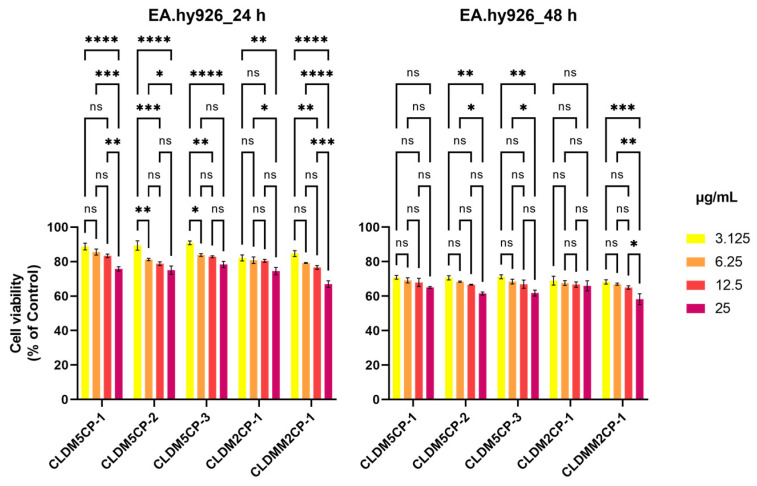
Comparative analysis of the EA.hy926 human endothelial cell viability influenced by 24 and 48 h treatment with magnetic cationic liposomes, in different concentrations. Statistical significance was determined by Tukey post hoc multiple comparison test (ns—nonsignificant, * *p* ≤ 0.05, ** *p* ≤ 0.01, *** *p* ≤ 0.001, **** *p* ≤ 0.0001).

To determine the statistical differences of the dexamethasone-loaded magnetic cationic liposome concentrations, a post hoc test was applied to both cell lines. Thus, according to the Tukey multiple comparison test, significant differences regarding the decrease in EA.hy296 endothelial cell viability were reported between the minimum and maximum concentrations studied, after 24 h of treatment, in the case of all liposomes tested (Figure 16), except the CLDM2CP-1 variant, where insignificant or weakly significant decreases in cell viability were recorded (* *p* ≤ 0.05 and ** *p* ≤ 0.01). It can be noted that the CLDM2CP-1 variant causes intensely significant differences (**** *p* ≤ 0.0001) when comparing the first two concentrations (3.125 and 6.25 µg/mL) with the maximum one (25 µg/mL). Interestingly, after 48 h of treatment, the decrease in cell viability does not vary significantly from one concentration to another, for all variants, although the percentage of cell viability is significantly lower compared to that obtained at 24 h. This response may suggest the slow and uniform release of dexamethasone from the liposomes. Once the drug has been released, it accumulates in the cells, inducing a certain cytotoxicity threshold that can be reached without the requirement of significant drug dose increasing, but only an extension of the exposure time.

**Figure 17:**
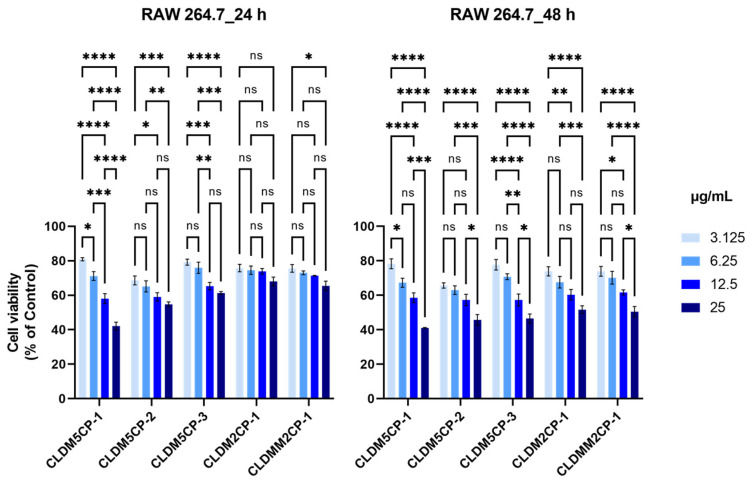
Comparative analysis of the RAW 264.7 macrophage viability influenced by 24 and 48 h treatment with magnetic cationic liposomes, in different concentrations. Statistical significance was determined by Tukey post hoc multiple comparison test (ns—nonsignificant, * *p* ≤ 0.05, ** *p* ≤ 0.01, *** *p* ≤ 0.001, **** *p* ≤ 0.0001).

Macrophage cells, key elements of the immune system, showed an intense response to the application of the newly synthesized liposomes, statistical differences with respect to the decrease in cell viability being recorded both after 24 h and after 48 h of treatment. At 24 h of exposure, significant differences were reported for the CLDM2CP-1, CLDM2CP-2, and CLDM2CP-3 variants. Prolonging the interaction of cells with dexamethasone-loaded magnetic cationic liposomes up to 48 h is associated with extensive cytotoxic phenomena, statistically significant (*p* ≤ 0.0001) for all variants, comparing the minimum concentration (3.125 µg/mL) with the maximum (25 µg/mL) (Figure 17).

Our results demonstrate that the mode of action of the newly synthesized magnetic cationic liposomes loaded with dexamethasone is specific to the cell type, with each cell line reacting differently to their action, there being a much more intense response in the cell line RAW 264.7. The encapsulation of dexamethasone in different PEG-liposomes significantly modifies the response of various primary human cells, leading to an intense cytotoxic response for certain cells (human primary macrophages or fibroblasts) and a non-cytotoxic response for other cell types (primary murine hepatocytes or endothelial cells) [81].

## 3. Materials and Methods

### 3.1. Materials

The tested nanoparticles were represented by four nanocapsule samples (OCNPM-1, OCNPM-1I, OCNPM-3, and OCNPM-3I) and five liposome samples (CLDM5CP-1, CLDM5CP-2, CLDM5CP-3, CLDM2CP-1, and CLDMM2CP-1), tested in vitro on two cell lines, at different doses and time intervals.

Biological material was represented by mouse macrophages RAW 264.7 (ATCC TIB-71) and human endothelial cells EA.hy926 (ATCC CRL-2922). Both cell lines were cultured in Dulbecco’s Modified Growth Medium (DMEM, PAN-Biotech GmbH, Aidenbach, Germany), supplemented with 10% fetal bovine serum (FBS, Euroclone S.p.A., Milano, Italy) and 1% antibiotic solution (Capricorn Scientific GmbH, Ebsdorfergrund, Germany), meaning penicillin 100 μg/mL and streptomycin 100 IU/mL, in an incubator (Binder GmbH, Tuttlingen, Germany) at 37 °C, in a humidified environment, with 5% CO_2_ [82].

#### Peptide-Functionalized Magnetic Nanocapsule and Liposome Preparation

Nanocapsules were prepared by the interfacial condensation method using two natural polymers (oligochitosan and carboxymethyl chitosan functionalized with peptides) and a synthetic polymer (poly NVPAI). Briefly, the organic copolymer solution (poly (NVPAI) dissolved in acetone and DMSO) was introduced dropwise into the aqueous solution containing the mixture of OCS, P1-CC, and P2-CC. The magnetic nanoparticles were introduced either in the aqueous solution (which forms the membrane of the nanocapsules) or in the organic solution (which forms the core of the nanocapsules). The amount of poly(NVPAI) was kept constant (250 mg) and the percentage of magnetic nanoparticles was 70% compared to the amount of copolymer (175 mg). In the case of samples OCNPM-1 and OCNPM-3, the magnetic particles were introduced into the core of the nanocapsules (in the organic phase), whereas in the case of OCNPM-1I and OCNPM-3I, the magnetic particles were loaded into the membrane of the nanocapsules (in the aqueous phase). The experimental program for the preparation of nanocapsules is presented in Table 7.

Liposomes were obtained from Egg Yolk Phospholipids (EPC), DOTAP (cationic lipid), and cholesterol (Chol) in different molar ratios, using the film hydration method followed by sonication. The experimental program for obtaining the liposomes is presented in Table 8.

Dexamethasone phosphate was encapsulated in the liposomes during the preparation process. The encapsulation degree was determined spectrophotometrically after the membrane of the liposomes was destroyed using a solution of 2% Triton x in water. It should also be mentioned that the degree of encapsulation with the anti-inflammatory drug was determined before the liposomes were coated with chitosan. For the preparation of chitosan-coated liposomes, a mixture of CMCS + CS-P1 + CS-P2 (concentration 0.5% in double distilled H_2_O) was added dropwise to the liposome suspension under magnetic stirring at room temperature for 2 h. The magnetic liposomes were prepared by film hydration (lipidic film was hydrated with a magnetic nanoparticle aqueous suspension or magnetic nanoparticles were introduced into the lipid solution prior to film formation) followed by sonication.

### 3.2. Methods

#### 3.2.1. Nanocarrier Physicochemical Characterization

Mean diameter and zeta potential of the nanocarriers (liposomes/nanocapsules) were measured using a Zetasizer Nano ZS from Malvern Panalytical. The release kinetics of dexamethasone was evaluated in phosphate buffer medium (PBS) (pH 7.4), using equipment from Agilent Dissolution Apparatus 708-DS equipped with a Sampling Station, 850-DS. A total of 200 mL of PBS was added to the small volume vessels, the temperature in the water bath was set to 37 °C, and the stirring of the paddles was set to 50 rpm. A total of 5 mL of the drug-loaded nanocarrier suspension was placed in cellulose dialysis tubes and the well-sealed tubes were immersed in the buffer solution. The drug concentration at the set times was determined spectroscopically (UV-VIS spectrophotometer Nanodrop One, λ = 242 nm).

#### 3.2.2. In Vitro Cell Viability Determination

The impact of the studied nanostructures on cell viability was assessed by the MTT method, a colorimetric method with 3-(4,5-dimethylthiazol-2-yl)-2,5-diphenyl tetrazolium bromide (MTT) [83,84,85]. This technique is based on the ability of mitochondrial dehydrogenases from living cells to transform the yellow water-soluble substrate (MTT) into dark blue, water-insoluble formazan. The total quantity of formazan is proportional to the number of living cells [86]. The EA.hy926 cells were detached with trypsin/EDTA, and the RAW 264.7 cells were scraped, then counted and resuspended in 96-well microplates (8 × 10^3^ cells/well for EA.hy926 cells and 5 × 10^3^ cells/well for RAW 264.7 cells), then maintained at the same temperature and humidity conditions. After 24 h, when the monolayer was formed, the cells were treated for 24 and 48 h with the nano-compounds, and added to the final culture medium (300 µL/well). The doses were 3.125, 6.25, 12.5, and 25 µg/mL. After treatment, cells were subjected to the processing steps of the MTT assay and absorbance was measured at 570 nm using the Biochrom EZ Read 400 automated microplate reader. Cell viability was calculated based on the following formula:

% Cell viability = A_sample_/A_control_ × 100

where:

A_sample_ = absorbance of the sample

A_control_ = absorbance of the control

#### 3.2.3. IC_50_ Calculation

IC_50_ values (sample concentration causing 50% inhibition of cell viability) of the newly synthesized nano-compounds were calculated using polynomial graphs in Excel software 2010.

#### 3.2.4. Cell Morphology Assay

A Nikon Eclipse TS100 inverted microscope supplied with an MshOt MS60 digital camera (Nikon, Tokyo, Japan) was used to evaluate the interaction of the different nanocarriers with EA.hy926 endothelial cells. Photographs were taken with a 10× objective, for each tested concentration, both after 24 and after 48 h of treatment, to identify the cytomorphological changes induced by the studied systems.

#### 3.2.5. Statistical Analysis

The results of the in vitro research were rendered as the mean of triplicate ± standard error (SE). The difference between mean values for each index was assessed using Student’s *t* test [87]. Probability values of less than 0.05 (*p* < 0.05) were considered statistically significant. The three-way ANOVA analysis was performed using Mathematica (version 14, Wolfram Research, Champaign, IL, USA) [88].

## 4. Conclusions

The results of the in vitro experiments performed on RAW 264.7 and EA.hy926 cells revealed that the nanocapsules based on chitosan, poly (NVPAI), and peptides, loaded with magnetic nanoparticles, are weakly cytotoxic for both cell lines. The magnetic cationic liposomes loaded with dexamethasone and coated with peptide-functionalized oligochitosan (CLDM5CP-1, CLDM5CP-2, and CLDM5CP-3) displayed a moderate cytotoxic action for both cell lines.

Regarding cellular internalization, the penetration of the nanocapsules through the cell membrane is more intense at the low used concentrations, due to the increase in the dose and the extension of the treatment time, facilitating the agglomeration of the nanocarriers. Commonly, at highest dose, the endocytosis process can be observed, proven by the appearance of some vesicles near the cell membrane. For liposomes, the incorporation process appears as more or less aggregated droplets, depending on the concentration and the duration of the treatment.

Based on these detailed in vitro tests, it can be concluded that both types of nanocarriers are suitable as drug delivery systems.

## Figures and Tables

**Table 1:** Mean diameter and zeta potential of magnetic nanocapsules.

Sample Code	Mean Diameter (Nm) in Distilled Water	ZP (mV) in PBS at pH 7.4
OCNPM-1	1075 ± 44	−30.1 ± 0.2
OCNPM-1I	1127 ± 53	−18.69 ± 1.53
OCNPM-3	923 ± 16	−24.8 ± 0.5
OCNPM-3I	995 ± 48	−19.93 ± 0.24

**Table 2:** The average size, the zeta potential, and the drug encapsulation degree for the obtained liposomes.

Sample Code	Mean Diameter (nm)	Zeta Potential (mV)	Drug Encapsulated (mg)
CLDM5CP-1	307 ± 8	+27 ± 1.4	2.29 ± 0.60
CLDM5CP-2	272 ± 2	+22 ± 0.3	2.20 ± 0.48
CLDM5CP-3	268 ± 3	+22 ± 0.4	2.10 ± 0.58
CLDM2CP-1	291 ± 24	+18 ± 0.6	2.15 ± 0.55
CLDMM2CP-1	260 ± 31	+16 ± 0.2	2.24 ± 0.070

**Table 3:** Inhibition concentration (IC_50_) values on RAW 264.7 and EA.hy926 cell lines after 24 and 48 h treatment with chitosan-based nanocapsules.

Cell Line	Variants	IC_50_ 24 h (µg/mL)	IC_50_ 48 h (µg/mL)
RAW 264.	OCNPM-1	76.627	67.407
	OCNPM-1I	74.122	68.560
	OCNPM-3	76.662	74.202
	OCNPM-3I	70.970	70.573
EA.hy926	OCNPM-1	63.224	67.516
	OCNPM-1I	79.923	65.997
	OCNPM-3	73.205	48.173
	OCNPM-3I	83.730	59.316

**Table 4:** Inhibition concentration (IC_50_) values on RAW 264.7 and EA.hy926 cell lines after 24 and 48 h treatment with magnetic cationic liposomes loaded with dexamethasone.

Cell Line	Variants	IC_50_ 24 h (µg/mL)	IC_50_ 48 h (µg/mL)
RAW 264.7	CLDM5CP-1	24.469	21.289
	CLDM5CP-2	38.178	22.841
	CLDM5CP-3	38.951	23.619
	CLDM2CP-1	59.683	26.282
	CLDMM2CP-1	54.498	25.195
EA.hy926	CLDM5CP-1	61.676	58.605
	CLDM5CP-2	60.085	51.611
	CLDM5CP-3	65.704	51.613
	CLDM2CP-1	66.470	62.637
	CLDMM2CP-1	47.979	47.064

**Table 5:** Results and summary interpretation of ANOVA for nanocapsules.

Factor (s)	*p*-Value			
	RAW 264.7 Cell Line		EA.hy926 Cell Line	
	24 h	48 h	24 h	48 h
MR	0.002	0.002	0.003	0.002
L	0.015	0.007	0.489	0.017
C	1.544 × 10^−6^	11.879 × 10^−6^	0.0002	4 × 10^−8^
L and MR	0.015	0.898	0.890	0.232
C and MR	0.771	0.042	0.518	0.0004
C and L	0.030	0.115	0.077	0.034
MR and L and C	0.151	0.449	0.388	0.075

**Table 6:** Results and summary interpretation of ANOVA for liposomes.

Factor (s)	*p*-Value			
	RAW 264.7 Cell Line		EA.Hy926 Cell Line	
	24 h	48 h	24 h	48 h
MR	0.815	0.612	0.011	0.661
MLs	0.011	0.118	0.003	0.004
C	51.70 × 10^−6^	1.23 × 10^−8^	6.00 × 10^−7^	9.15 × 10^−7^
C and MR	0.963	0.695	0.995	0.164
C and MLs	0.018	0.267	0.446	0.237

**Table 7:** Experimental program and codification of synthesized samples.

Sample Code	NH_2_ Groups of OCS/Anhydride Cycles of Poly(NVPAI) (Moles/Moles)	Mixture of OCS, P1-CC and P2-CC		
		OCS (%)	P1-CC (%)	P2-CC (%)
OCNPM-1	0.5/1	90	5	5
OCNPM-1I	0.5/1	90	5	5
OCNPM-3	1.5/1	97.5	1.25	1.25
OCNPM-3I	1.5/1	97.5	1.25	1.25

P1-CC: carboxymethyl chitosan functionalized with TAT peptide; P2-CC: carboxymethyl chitosan functionalized with Tet 1 peptide. The concentration of poly(NVPAI) in the organic solvent was 1% (*w*/*v*).

**Table 8:** The experimental program for obtaining liposomes and the sample code.

Sample Code	EPC (mg)	Chol (mg)	DOTAP (mg)	Dexamethasone Phosphate (mg)	Magnetic NPs (mg)	CMCS + CS-P1 + CS-P2 * (0.5%)/Liposomes Suspension (*v*/*v*)
CLDM50CP-1	22	2	1	25	12.5 in drug solution	2/1
CLDM50CP-2	21	2	2	25		2/1
CLDM50CP-3	19	4	2	25		2/1
CLDM2CP-1	22	2	1	25	10 in drug solution	2/1
CLDMM2CP-1	22	2	1	25	10 in lipids solution	2/1

* CMCS/(CS-P1 + CS-P2) = 9/1 (*w*/*w*).

## Data Availability

Data will be made available on request.
